# Does chemotherapy regimen matter for first-line immunochemotherapy in low PD-L1-expressing esophageal squamous cell carcinoma? A systemic review and meta-analysis

**DOI:** 10.1007/s10388-025-01167-y

**Published:** 2025-11-10

**Authors:** Jhe-Cyuan Guo, Pei-Shan Lin, Wen-Yi Shau, Yu-Yun Shao, Hung-Yang Kuo, Chi-Ling Chen, Chih-Hung Hsu

**Affiliations:** 1 Department of Medical Oncology, National Taiwan University Cancer Center, Taipei, Taiwan; 2 Department of Oncology, National Taiwan University Hospital, Taipei, Taiwan; 3 Department of Surgery, National Taiwan University Hospital, Taipei, Taiwan; 4 Graduate Institute of Clinical Medicine, College of Medicine, National Taiwan University, Taipei, Taiwan; 5 Graduate Institute of Oncology, College of Medicine, National Taiwan University, No.1, Section 1, Jen Ai Road, Taipei, Taiwan 10051; 6 Graduate Institute of Epidemiology and Preventive Medicine, College of Public Health, National Taiwan University, Taipei, Taiwan; 7 Hepatitis Research Center, National Taiwan University Hospital, Taipei, Taiwan

**Keywords:** Esophageal squamous cell carcinoma, Chemotherapy, PD-L1 expression, Immune checkpoint inhibitor

## Abstract

Anti-PD-1 therapy plus chemotherapy (immunochemotherapy) has become standard first-line treatment for high PD-L1-expressing advanced esophageal squamous cell carcinoma (ESCC). Benefits of immunochemotherapy for low PD-L1-expressing ESCC remain debatable. The Cochrane, PubMed, and Embase databases were systemically searched from inception till 10 August 2024. Randomized trials comparing first-line immunochemotherapy with chemotherapy in ESCC were identified and evaluated association of platinum plus paclitaxel (TP) or fluoropyrimidine (PF) chemotherapy regimen, stratified by PD-L1 expression levels, with progression-free survival (PFS) and overall survival (OS) benefits. Pooled study-level hazard ratios (HRs) with 95% confidence intervals (CIs) for PFS and OS were calculated with a random-effects model. Eight studies involving 4733 participants were included. In high PD-L1 group, PFS (HR of TP: 0.56 [95% CI, 0.45–0.69] vs HR of PF: 0.53 [95% CI, 0.45–0.62]) and OS benefits (HR of TP: 0.60 [95% CI, 0.46–0.78] vs HR of PF: 0.59 [95% CI, 0.50–0.69]) did not significantly differ between two regimen subgroups (*P* = 0.75 and 0.90, respectively). In low PD-L1 group, TP regimen was associated with a significantly greater PFS benefit than PF regimen (HR of TP: 0.59 [95% CI, 0.48–0.74] vs HR of PF: 0.82 [95% CI, 0.72–0.94]; *P* = 0.01) and TP regimen trended to associate with greater OS benefit over PF regimen (HR of TP: 0.72 [95% CI, 0.55–0.93] vs HR of PF: 0.84 [95% CI, 0.72–0.97]; *P* = 0.32). In patients with low PD-L1-expressing advanced ESCC, immunochemotherapy with TP may confer a greater PFS benefit than that with PF.

**Supplementary Information:**

The online version contains supplementary material available at 10.1007/s10388-025-01167-y.

## Background

In 2022, esophageal cancer (EC) was the 11th most commonly diagnosed cancer and the 7th leading cause of cancer-related mortality worldwide [1]. Approximately 85% of EC cases were esophageal squamous cell carcinoma (ESCC), the predominant histological subtype, whereas the remaining 15% were esophageal adenocarcinoma (EAC) and other rare subtype [2]. ESCC and EAC exhibit distinct geographical distributions, risk factors, and genetic alterations [3, 4].

Before the advent of modern immunotherapy, chemotherapy was the only systemic treatment modality for patients with metastatic, recurrent, or unresectable locally advanced ESCC. Platinum-based chemotherapy was considered as the standard first-line therapy for advanced ESCC [5]. The median survival for patients with advanced ESCC ranged between 8 and 10 months, with only a small percentage of patients surviving beyond 2 years [6, 7].

Immune checkpoint inhibitors (ICIs), particularly anti-programmed death 1 (PD-1) and programmed cell death ligand 1 (PD-L1) therapies, have transformed the treatment landscape for various cancer types, including EC and ESCC [8, 9]. Four phase III randomized controlled trials (RCTs) compared anti-PD-1 therapy with second-line systemic chemotherapy in patients with advanced ESCC who previously failed platinum-based chemotherapy and had not had prior anti-PD-1/PD-L1 therapy. These studies have demonstrated anti-PD-1 therapy to be a preferred second-line therapy, with a significant improvement of the median overall survival (OS) from 6.2 to 8.4 months to 8.2 to 10.9 months and a hazard ratio (HR) of 0.70 to 0.78 [10–13]. Further, 8 phase III RCTs evaluated the combination of anti-PD-1/PD-L1 therapy and chemotherapy as first-line systemic therapy [14–26]. Despite differences in the specific ICIs, chemotherapy backbones, and study designs employed, all 8 trials revealed improved treatment outcomes when anti-PD-1/PD-L1 therapy was added to first-line chemotherapy. The median OS in patients with advanced ESCC increased from 9.8 to 12.5 months to 12.6 to 17.2 months, with HRs of 0.58 to 0.74. Additionally, the combination of anti-PD-1/PD-L1 therapy and chemotherapy resulted in improved progression-free survival (PFS), with an increase in the median PFS from 5.3 to 5.8 months to 5.7 to 7.3 months, with HRs of 0.56 to 0.81, along with a higher objective response rate (ORR) (increase from 27.0%−62.1% to 43.8%−72.1%). Collectively, these 8 trials support the use of anti-PD-1/PD-L1 therapy in conjunction with chemotherapy as a new standard first-line systemic therapy for advanced ESCC [14–26].

The expression level of PD-L1 within the tumor microenvironment, which is a target for PD-1/PD-L1 pathway blockade, has been extensively investigated as a predictive biomarker of the efficacy of anti-PD-1/PD-L1-based ICIs across various cancer types. The results of studies on the topic, however, have varied. In ESCC, several studies demonstrated patients with high PD-L1 expression obtained more benefit from anti-PD-1/PD-L1 therapy plus chemotherapy compared with chemotherapy alone than patients with low PD-L1 expression [14–19, 21, 25, 26]. The European Society for Medical Oncology practice guideline published in 2022 and the American Society of Clinical Oncology guideline published in 2023 stratify first-line therapy recommendations on the basis of PD-L1 expression levels [27, 28]. For individuals with a PD-L1 combined positive score (CPS) of ≥ 10, the recommended treatment is pembrolizumab in combination with platinum and fluoropyrimidine. Alternatively, for patients with a PD-L1 tumor proportion score (TPS) of ≥ 1%, the recommended treatment is either nivolumab plus platinum and fluoropyrimidine or nivolumab plus ipilimumab. For cases with low PD-L1 expression levels, both guidelines recommend the use of platinum and fluoropyrimidine alone [27, 28]. However, other treatment guidelines, such as the Japanese Esophageal Society in their 2022 EC practice guidelines, advocate for the combination of anti-PD-1/PD-L1 therapy with cisplatin and 5-fluorouracil (5-FU) as preferred first-line therapy for advanced ESCC, regardless of PD-L1 expression levels [29]. In general, the benefit of adding anti-PD-1/PD-L1 therapy to chemotherapy as first-line treatment for patients with low PD-L1-expressing advanced ESCC remains a topic of debate.

Several meta-analyses have been conducted to evaluate the effectiveness of anti-PD-1/PD-L1 therapy in combination with chemotherapy as a first-line treatment for advanced EC and ESCC [30–32]. These analyses have consistently demonstrated survival benefits associated with the addition of anti-PD-1/PD-L1 therapy to chemotherapy in patients with advanced ESCC exhibiting high PD-L1 expression levels. However, the results regarding the benefit of adding anti-PD-1/PD-L1 therapy to chemotherapy in patients with advanced ESCC exhibiting low PD-L1 expression levels have been inconsistent [30, 31]. The current meta-analysis represents the most comprehensive effort in understanding such benefits to date; it included all 8 RCTs that have been published on this topic. This study not only addressed how PD-L1 expression levels influence the efficacy of adding anti-PD-1/PD-L1 therapy to first-line chemotherapy in patients with advanced ESCC, but also investigated whether different chemotherapy regimens affect the benefits conferred by the addition of anti-PD-1/PD-L1 therapy.

## Methods

### Search strategy for and systematic review of eligible studies

The PICO framework was employed to select eligible articles on the basis of the following: (1) population: Patients with unresectable locally advanced or metastatic ESCC who had not received prior treatment for advanced disease. (2) Intervention: Treatment with anti-PD-1/PD-L1 therapy in combination with chemotherapy. (3) Control: Treatment with chemotherapy alone. (4) Outcome: Assessment of OS and PFS.

Searches were conducted of the Cochrane, PubMed, and Embase databases on August 10, 2024 (P.-S. L. and J.-C. G.). The search terms were as follows: (first line) AND [(esophageal squamous cell carcinoma) OR (oesophageal squamous cell carcinoma) OR (esophageal cancer) OR (oesophageal cancer)] AND [(PD-1) OR (PD-L1)] AND (chemotherapy) AND (overall survival). The systematic review was conducted in accordance with PRISMA guidelines and included the following steps: (1) removal of duplicate studies, (2) review of study titles and abstracts to ensure inclusion of studies relevant to the research question and exclusion of those deemed nonrelevant, (3) exclusion of studies without full text before full-text screening, and (4) review of full-text studies for eligibility by employing the established PICO inclusion criteria.

### Data extraction and quality assessment

The data extracted from each study comprised the study name, treatment combinations used in the study group, chemotherapy regimen, and number of participants in each group and subgroup. Clinical data included HRs for PFS and OS along with their corresponding 95% confidence intervals (CIs).

The Cochrane Collaboration tool was employed for quality assessment. Bias was evaluated by assessing individual elements within the following domains: randomization process, deviations from intended interventions, missing outcome data, measurement of the outcome and selection of the reported result. Each domain was rated on the basis of the level of concern: no information, some concerns, high risk, or low risk of bias.

### Study characteristics and risk of bias

The current meta-analysis included 8 phase III RCTs which involved a total of 4733 participants. KEYNOTE-590 trial investigated pembrolizumab, enrolled patients with ESCC and adenocarcinoma of the esophagus and gastroesophageal junction [14, 15] The remaining studies—that is, CheckMate 648 (studying nivolumab) [16–18], ASTRUM-007 (studying serplulimab) [25], GEMSTONE-304 (studying sugemalimab) [26], ESCORT-1st (studying camrelizumab) [20, 21], JUPITER-06 (studying toripalimab) [24], ORIENT-15 (studying sintilimab) [22, 23], and RATIONALE-306 (studying tislelizumab) [19]—focused exclusively on patients with ESCC. All RCTs compared a combination of anti-PD-1/PD-L1 therapy and chemotherapy in the intervention arms against chemotherapy alone in the control arms. All studies investigated anti-PD-1 therapy, with the exception of GEMSTONE-304, which investigated sugemalimab, an anti-PD-L1 therapy. ORIENT-15 and RATIONALE-306 allowed investigators to select more than one chemotherapy regimen, whereas the other 6 RCTs investigated a specific chemotherapy regimen. The chemotherapy regimens across the trials included cisplatin/5-FU (in 6 studies), oxaliplatin/5-FU (in 1 study), cisplatin/capecitabine (in 1 study) and oxaliplatin/capecitabine (in 1 study), paclitaxel/cisplatin (in 4 studies), and paclitaxel/oxaliplatin (in 1 study). Across all trials, the combination of anti-PD-1/PD-L1 therapy with chemotherapy demonstrated superior efficacy over chemotherapy alone, as evidenced by improvements in both PFS and OS. Table 1 provides a detailed summary of the treatment regimens, the number of participants, and the efficacy outcomes, including the median durations of PFS and OS, and the corresponding HRs for each treatment arm in these trials.

**Table 1:** Summary of the trials included in the meta-analysis

Study	N		PD-1/PD-L1 inhibitor	Chemotherapy			mOS(m)[95%CI]		OS HR [95%CI]	mPFS(m)[95%CI]		PFS HR [95%CI]
	Exp	Control		5-fluorouracil	cisplatin	paclitaxel	Exp	Control		Exp	Control	
KEYNOTE-590-SCC	274	274	Pembrolizumab 200 mg on D1	800 mg/m^2^ on D1-5	80 mg/m^2^ on D1	NA	12.6 [10.2–14.3]	9.8 [8.6–11.1]	0.71 [0.60–0.85]	6.3 [6.2–6.9]	5.8 [5.0–6.1]	0.65 [0.54–0.78]
CheckMate 648	321	324	Nivolumab 240 mg on D1 & D15	800 mg/m^2^ on D1-5	80 mg/m^2^ on D1	NA	13.2 [11.1–15.7]	10.7 [9.4–12.1]	0.77 [0.65–0.92]	5.8 [5.5–7]	5.6 [4.3–5.9]	0.82 [0.68–1.00]
ASTRUM-007	368	183	Serplulimab at a dose of 3 mg/kg on D1	1200 mg/m^2^ on D1-2	50 mg/m^2^ on D1	NA	15.3 [14.0–18.6]	11.8 [9.7–14.0]	0.68 [0.53–0.87]	5.8 [5.7–6.9]	5.3 [4.3–5.6]	0.60 [0.48–0.75]
GEMSTONE-304	358	182	Sugemalimab 1200 mg on D1	800 mg/m^2^ on D1-4	80 mg/m^2^ on D1	NA	15.3 [13.3–17.1]	11.5 [9.9–13.4]	0.70 [0.55–0.90]	6.2 [5.7–6.9]	5.4 [4.9–5.8]	0.67 [0.54–0.82]
ESCORT-1st	298	298	Camrelizumab 200 mg on D1	NA	75 mg/m^2^ on D1	175 mg/m^2^ on D1	15.3 [12.8–17.3]	12.0 [11.0–13.3]	0.70 [0.58–0.84]	6.9 [5.8–7.4]	5.6 [5.5–5.7]	0.54 [0.45–0.65]
JUPITER-06	257	257	Toripalimab 240 mg on D1	NA	75 mg/m^2^ on D1	175 mg/m^2^ on D1	17 [14.0-NR]	11.0 [10.4–12.60]	0.58 [0.43–0.78]	5.7 [5.6–7]	5.5 [5.2–5.6]	0.58 [0.46–0.74]
ORIENT-15	341	349	Sintilimab 200 mg (≥ 60 kg) /3 mg/kg (< 60 kg) on D1	800 mg/m^2^ on D1-5	75 mg/m^2^ on D1	175 mg/m^2^ on D1	17.4 [16.0–19.8]	12.8 [11.3–14.5]	0.66 [0.55–0.79]	7.2 [7–9.6]	5.7 [5.5–6.8]	0.56 [0.46–0.68]
ORIENT-15 (PF)	20	23					NC [8.9-NC]	NC [3.3-NC]	0.31 [0.08–1.20]	5.8 [4-NC]	4.0 [1.3-NC]	0.55 [0.23–1.32]
ORIENT-15 (TP)	307	309					16.7 [14.8–21.7]	12.5 [10.4–15.7]	0.65 [0.52–0.80]	8.2 [7–9.7]	5.9 [5.5–6.9]	0.55 [0.45–0.67]
RATIONALE-306	326	323	Tislelizumab 200 mg on D1	750–800 mg/m^2^ on D1-5 (or capecitabine 1000 mg/m^2^ orally twice daily on D1-14)	60–80 mg/m^2^ (or oxaliplatin 130 mg/m^2^) on D1	175 mg/m^2^ on D1	17.2 [15.8–20.1]	10.6 [9.3–12.1]	0.66 [0.54–0.80]	7.3 [6.9–8.3]	5.6 [4.9–6.0]	0.62 [0.52–0.75]
RATIONALE-306 (PF)	147	146					20.4 [16.3–23.7]	10.6 [9–15.1]	0.66 [0.49–0.88]	7.0 [6.7–8.3]	5.5 [4.3–5.8]	0.66 [0.51–0.86]
RATIONALE-306 (TP)	179	177					15.8 [12.8–18.6]	10.6 [8.9–12.0]	0.69 [0.54–0.89]	8.2 [6.9–8.9]	5.6 [4.9–6.8]	0.57 [0.45–0.74]

*PF* fluoropyrimidine (5-fluorouracil or capecitabine) + platinum (cisplatin or oxaliplatin), *TP* paclitaxel + platinum (cisplatin or oxaliplatin), *NA* not applicable, *NC* not calculated, *NR* not reached

Seven of the 8 RCTs in this meta-analysis exclusively enrolled patients with ESCC. The KEYNOTE-590 trial included patients with both adenocarcinoma of the Siewert type 1 gastroesophageal junction and esophagus as well as ESCC. However, only data from KEYNOTE-590 that were specific to patients with ESCC were included in the current study [14, 15]. The CheckMate 648 trial comprised 3 arms: arm A received ipilimumab (a cytotoxic T lymphocyte associated protein [CTLA-4] inhibitor) plus nivolumab, arm B received nivolumab combined with chemotherapy, and arm C received chemotherapy alone. In the current study, only the data from arms B and C of CheckMate 648 were analyzed [16–18]. The RATIONALE-306 trial employed several chemotherapy combinations. These included platinum agents (cisplatin or oxaliplatin) combined with a fluoropyrimidine (5-fluorouracil or capecitabine), classified as PF regimens, and platinum combined with paclitaxel, classified as TP regimens [19]. The ASTRUM-007 study exclusively enrolled patients with a PD-L1 CPS of ≥ 1 [25]. The quality assessment results are presented in Figure S1.

### Statistical analysis

Statistical analysis was conducted by using the RStudio software (v4.3.2) and a random-effects model was employed. HRs for PFS and OS, along with their respective 95% CIs, were used to compare the treatments in both the meta-analysis and network meta-analysis. Three distinct criteria for scoring PD-L1 expression were adopted: TPS (≥ 1% versus < 1%), tumor area positivity (TAP, ≥ 10% versus < 10%), and CPS (≥ 10 versus < 10). Additionally, the efficacy of 2 chemotherapy regimens—TP (paclitaxel plus platinum [cisplatin or oxaliplatin]) and PF (fluoropyrimidine [5-fluorouracil or capecitabine] plus platinum [cisplatin or oxaliplatin])—was evaluated, with stratification by PD-L1 expression levels. This comprehensive analysis enabled a comparison of treatment regimens and classification on the basis of PD-L1 expression and chemotherapy regimens, providing a robust framework for interpreting the results within both the meta-analysis and network meta-analysis.

## Results

### Study selection

The electronic search yielded 555 potential articles. A total of 225 duplicate records were removed, and the titles and abstracts of the remaining 330 records were screened with consideration of the PICO criteria of this study. Subsequently, 301 articles were excluded from further consideration. After a full review of the remaining 29 articles, 21 were excluded for the following reasons: (1) inclusion of mixed data for multiple treatment lines (n = 2), (2) absence of HR data for OS (n = 4), (3) retrospective study design (n = 1), (4) use of obsolete data (n = 9), and (5) updated follow-up data from the same study (n = 5). Ultimately, 8 studies were deemed eligible for inclusion in the analysis, and for 4 studies—KEYNOTE-590, CheckMate 648, ESCORT-1st, and ORIENT-15—their most recent data were considered [14–26]. Figure S2 illustrates the selection process and the reasons for exclusion.

### Meta-analysis

**Fig. 1:**
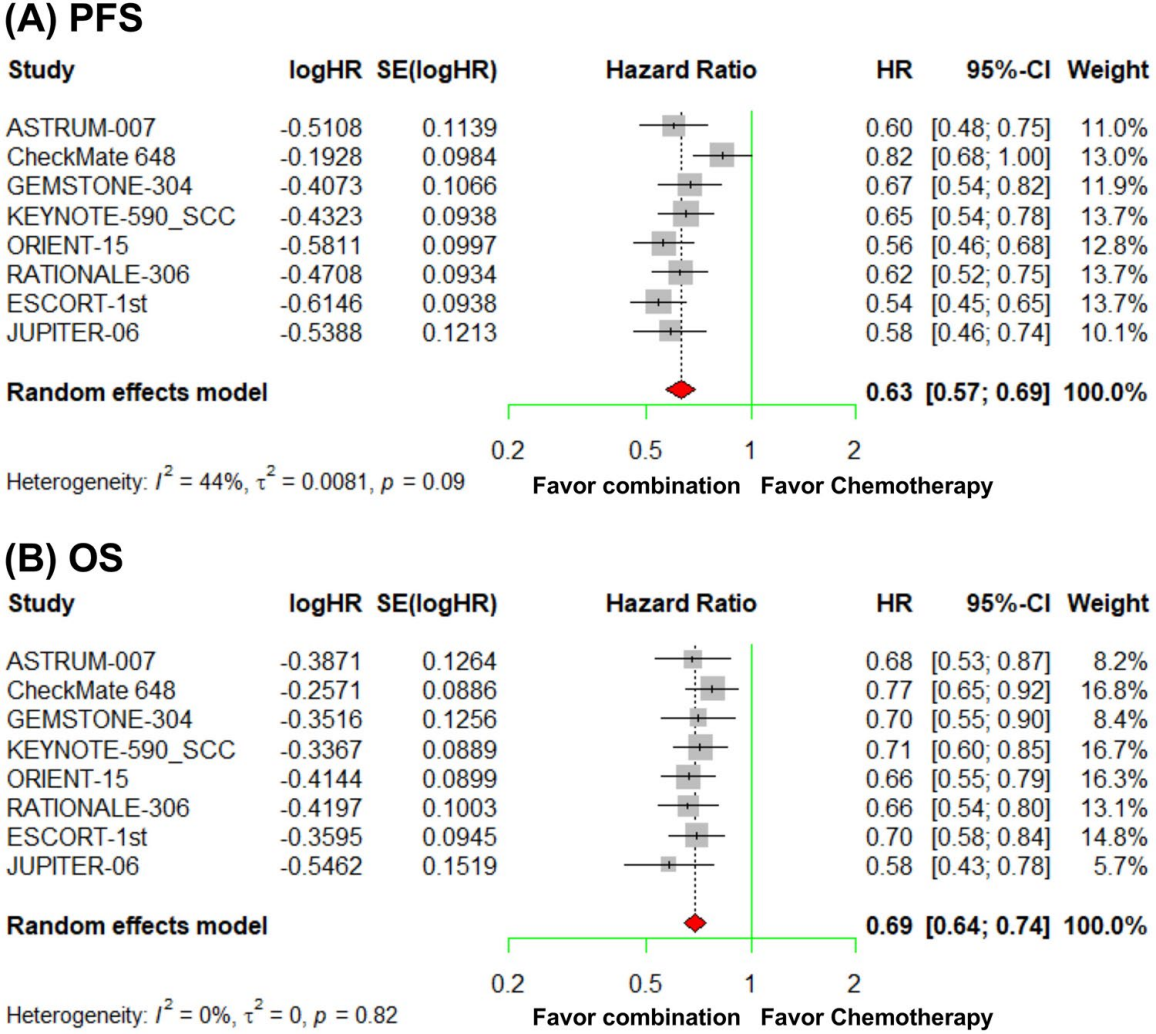
Forest plots from the meta-analysis illustrating the comparison of progression-free survival (PFS) (**a**) and overall survival (OS) (**b**) between anti-PD-1/PD-L1 therapy combined with chemotherapy and chemotherapy alone

The combination of anti-PD-1/PD-L1 therapy and chemotherapy, i.e., the immunochemotherapy, demonstrated significant superiority over chemotherapy alone in terms of survival outcomes. This was true for both PFS, for which the pooled HR was 0.63 (95% CI, 0.57–0.69), and for OS, for which the pooled HR was 0.69 (95% CI, 0.64–0.74; Fig. 1).

### One-level subgroup analysis by PD-L1 expression levels

Patients were stratified by PD-L1 expression levels into high and low expression groups. For PFS, the pooled analysis demonstrated that immunochemotherapy significantly outperformed chemotherapy alone, with a HR of 0.54 (95% CI, 0.49–0.60) in patients with high PD-L1 expression levels. By contrast, for patients with low PD-L1 expression, the HR was 0.71 (95% CI, 0.62–0.81). Additionally, for OS, patients with high PD-L1 expression had a HR of 0.60 (95% CI, 0.53–0.67) for immunochemotherapy versus chemotherapy alone. In the low PD-L1 expression group, the corresponding HR was 0.78 (95% CI, 0.70–0.87). Subgroup analysis revealed a statistically significant difference in both PFS (*P* < 0.01) and OS (*P* < 0.01) between patients with high and low PD-L1 expression.

**Fig. 2:**
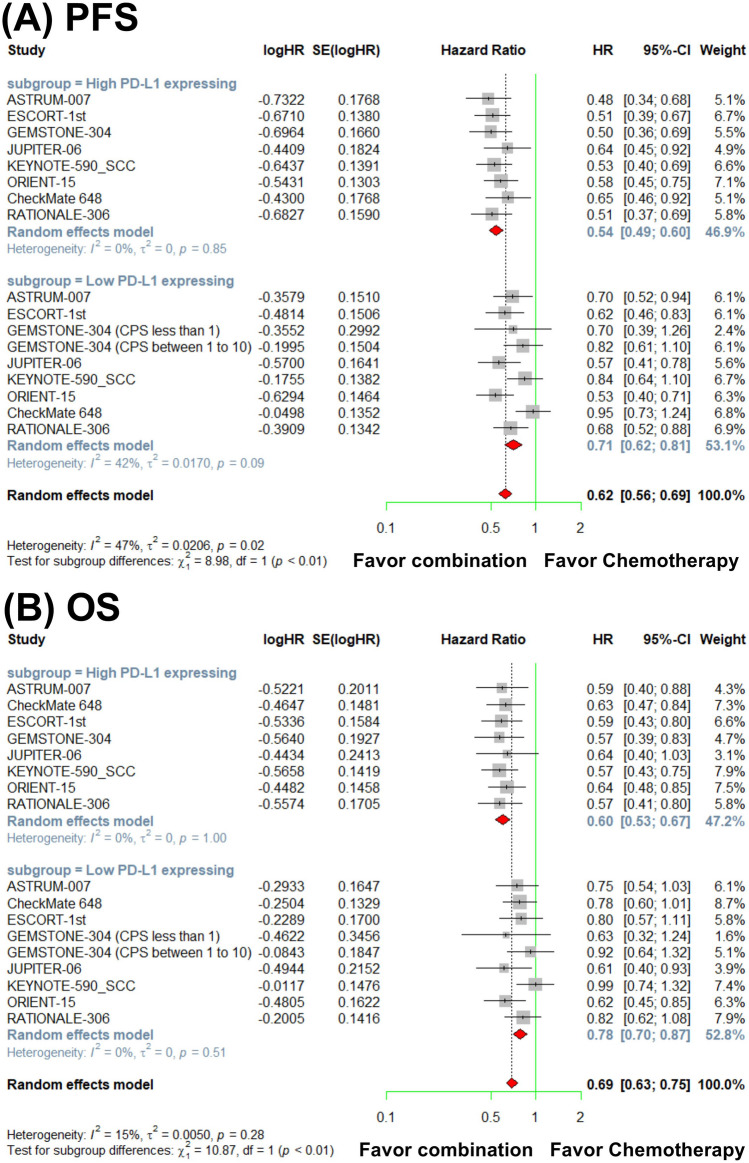
Forest plots from the meta-analysis illustrating progression-free survival (PFS) (**a**) and overall survival (OS) (**b**) in a comparison of anti-PD-1/PD-L1 therapy combined with chemotherapy and chemotherapy alone, stratified by PD-L1 expression level (high PD-L1 expression: CPS ≥ 10, TAP ≥ 10, or TPS ≥ 1; low PD-L1 expression: CPS < 10, TAP < 10, or TPS < 1)

These findings indicate that the survival benefit derived from adding anti-PD-1/PD-L1 therapy to chemotherapy is more pronounced in patients with high PD-L1 expression levels than in those with low PD-L1 expression levels (Fig. 2) (Table S1).

### One-level subgroup analysis by chemotherapy regimens

Analysis of the impact of chemotherapy regimens on PFS benefits revealed that patients treated with immunochemotherapy with PF regimen exhibited a HR of 0.68 (95% CI, 0.62–0.75) compared with PF chemotherapy alone. Moreover, those treated with immunochemotherapy with TP regimen exhibited a HR of 0.56 (95% CI, 0.50–0.62) compared with TP chemotherapy alone. Regarding OS, immunochemotherapy with PF regimen yielded a HR of 0.72 (95% CI, 0.65–0.79) compared with PF chemotherapy alone, and immunochemotherapy with TP regimen yielded an HR of 0.66 (95% CI, 0.59–0.74) compared with TP chemotherapy alone. A statistically significant difference in PFS benefit was observed in patient subgroups treated with PF and TP chemotherapy (*P* < 0.01), whereas no significant difference in OS benefit was noted in the patient subgroups treated with these 2 chemotherapy regimens (*P* = 0.32).

**Fig. 3:**
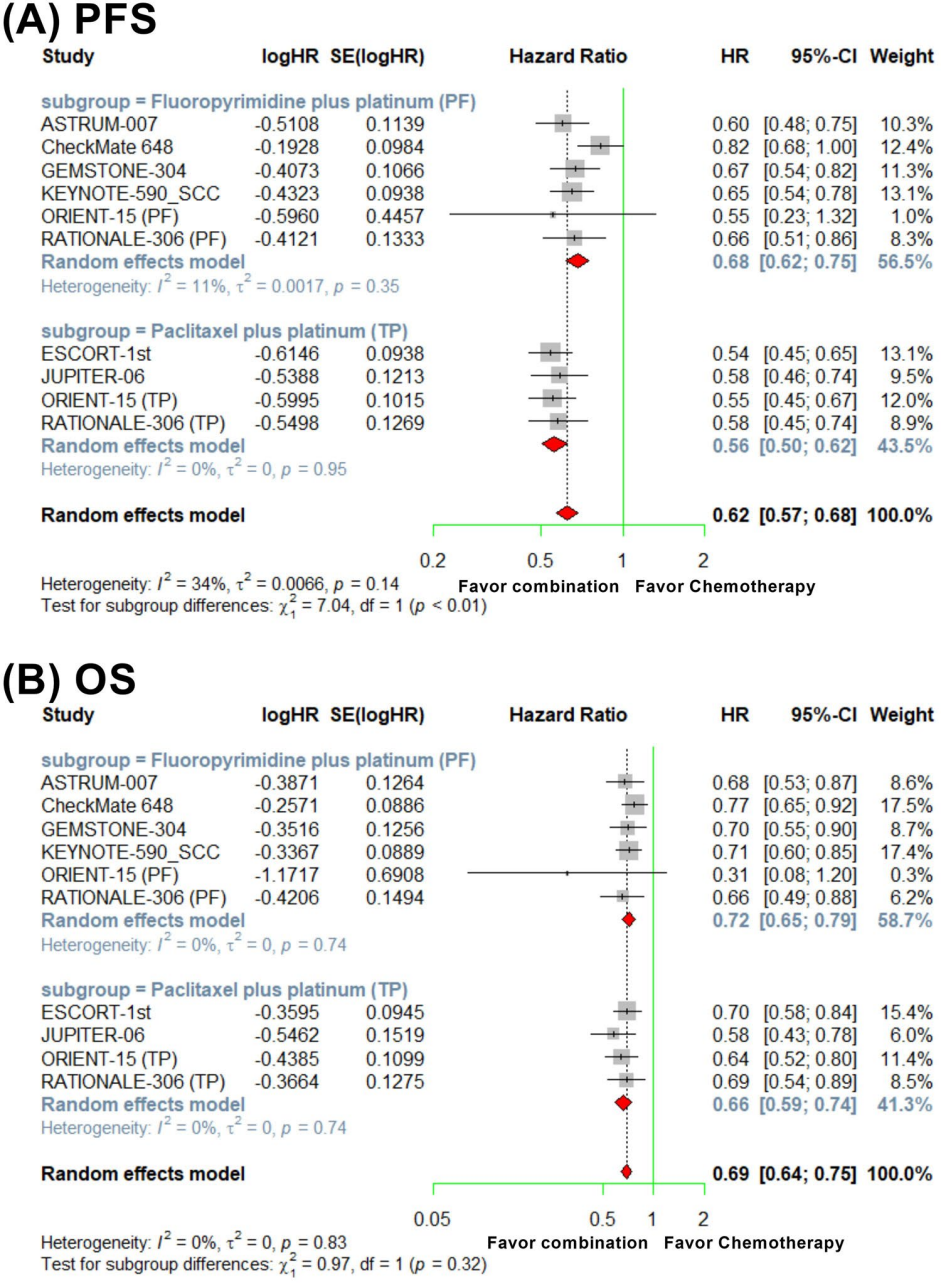
Meta-analysis forest plots for progression-free survival (PFS) (**a**) and overall survival (OS) (**b**) in a comparison of anti-PD-1/PD-L1 therapy combined with chemotherapy and chemotherapy alone, stratified by chemotherapy regimen (*PF* fluoropyrimidine plus platinum, *TP* paclitaxel plus platinum)

These findings indicate that the PFS benefit and OS benefit associated with the addition of anti-PD-1/PD-L1 therapy to chemotherapy were seen in both chemotherapy regimens although there might be a more pronounced PFS benefit seen in patients treated with TP regimen than those treated with PF regimen (Fig. 3) (Table S2).

### Two-level subgroup analysis cross PD-L1 expression levels and chemotherapy regimens

**Fig. 4:**
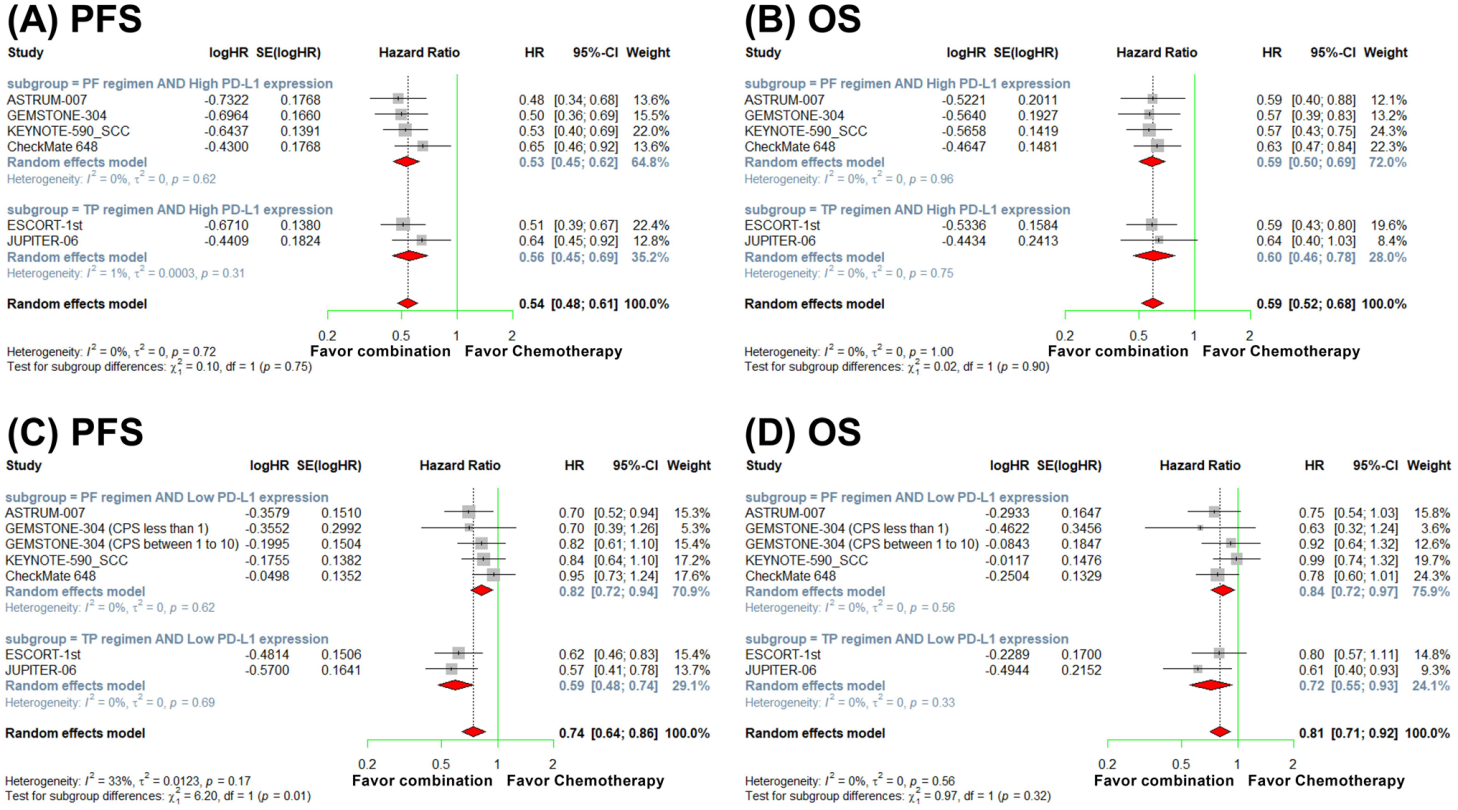
Meta-analysis forest plots for progression-free survival (PFS) and overall survival (OS) for patients receiving anti-PD-1/PD-L1 therapy combined with chemotherapy versus chemotherapy alone, stratified by PD-L1 expression level (high PD-L1 expression: CPS ≥ 10, TAP ≥ 10, or TPS ≥ 1; low PD-L1 expression: CPS < 10, TAP < 10, or TPS < 1) and chemotherapy regimen (*PF* fluoropyrimidine plus platinum, *TP* paclitaxel plus platinum) in the high PD-L1 expression (**a** and **b**) and low PD-L1 expression subgroups (**c** and **d**)

We examined the effects of chemotherapy regimens on survival benefits of immunochemotherapy over chemotherapy in patients with different PD-L1 expression levels. In the high PD-L1 expression group, immunochemotherapy with PF regimen resulted in a significant PFS (HR, 0.53; 95% CI, 0.45–0.62) and OS benefits (HR, 0.59; 95% CI, 0.50–0.69) compared with PF chemotherapy alone. The immunochemotherapy with TP regimen also yielded significant PFS (HR, 0.56; 95% CI, 0.45–0.69) and OS (HR, 0.60; 95% CI, 0.46–0.78) benefits compared with TP chemotherapy alone. Notably, in patients with high PD-L1 expression levels, the PFS and OS benefit was were comparable between patients treated with PF chemotherapy and those receiving TP chemotherapy (*P* = 0.75 and *P* = 0.90, respectively; Fig. 4a and b) (Table S3).

In the low PD-L1 expression group, immunochemotherapy with PF regimen yielded modest PFS (HR, 0.82; 95% CI, 0.72–0.94) and OS (HR, 0.84; 95% CI, 0.72–0.97) benefits compared with PF chemotherapy alone. Immunochemotherapy with TP regimen exhibited more pronounced PFS (HR, 0.59; 95% CI, 0.48–0.74) and OS (HR, 0.72; 95% CI, 0.55–0.93) benefits compared with TP chemotherapy alone. In the low PD-L1 expression group, the PFS benefit was significantly greater for patients treated with TP chemotherapy than for those treated with PF chemotherapy (*P* = 0.01) and the OS benefit was numerically in favor of TP chemotherapy, although this association did not reach statistical significance (*P* = 0.32; Fig. 4c and d) (Table S3).

These findings suggest that in patients with low PD-L1 expression, the addition of anti-PD-1/PD-L1 therapy to chemotherapy confers a more significant PFS benefit when such therapy is combined with the TP regimen relative to when it is combined with the PF regimen, indicating a significant differential effect between the chemotherapy regimens in this subgroup.

### Network meta-analysis

**Fig. 5:**
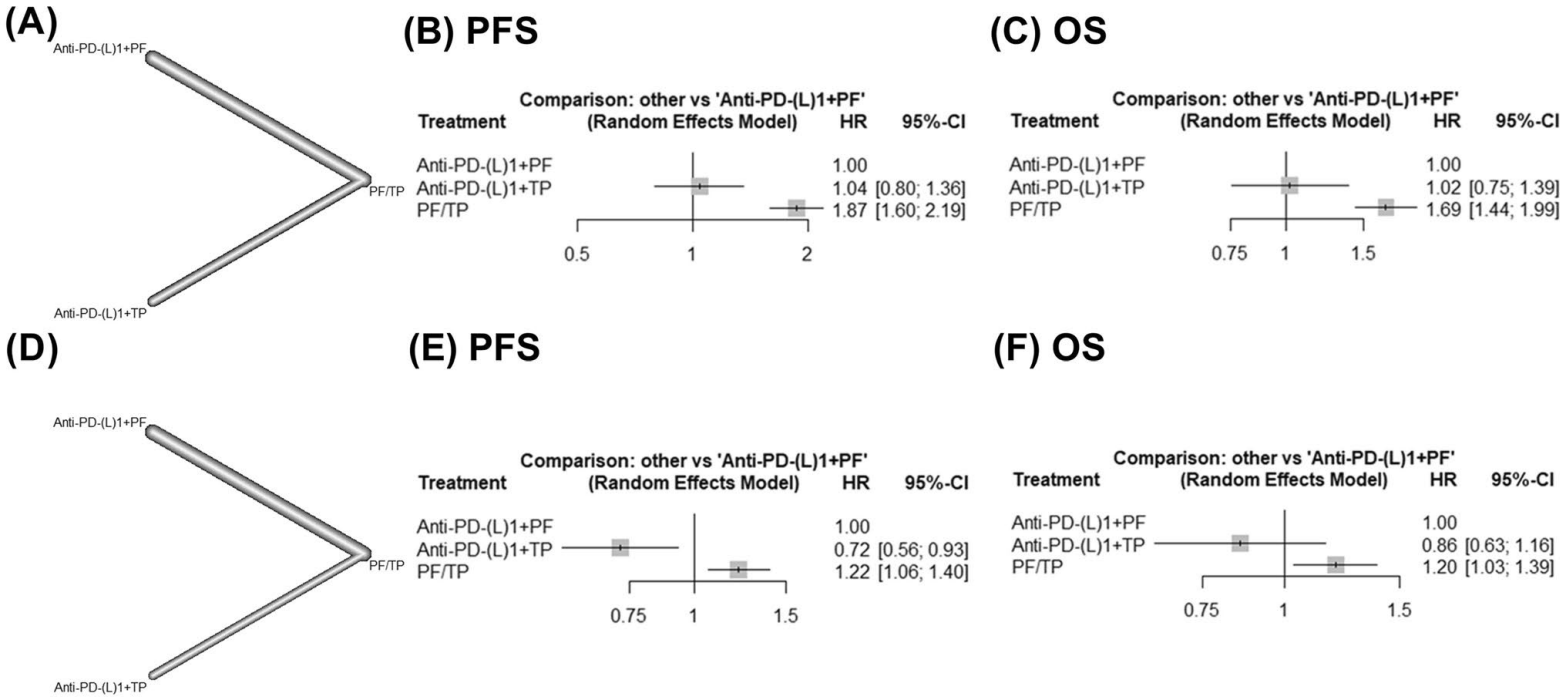
Network meta-analysis forest plots depicting progression-free survival (PFS) and overall survival (OS) in a comparison of anti-PD-1/PD-L1 therapy combined with paclitaxel plus platinum (TP) or fluoropyrimidine plus platinum (PF) in patients with high (**a**–**c**) and low (**d**–**f**) PD-L1 expression

A network meta-analysis was conducted to compare the efficacy of 3 treatment strategies: chemotherapy alone (either PF or TP regimen), immunochemotherapy with PF regimen, and immunochemotherapy with TP regimen among all studies except ORIENT-15 and RATIONALE-306. The results of an analysis of patients with high and low PD-L1 expression levels are presented in Fig. 5.

### Comparison of PFS and OS in patients with high PD-L1 expression levels by network meta-analysis

A network analysis focused on patients with high PD-L1 expression levels revealed that patients receiving immunochemotherapy with TP regimen demonstrated comparable PFS (HR, 1.04; 95% CI, 0.80–1.36) and OS (HR, 1.02; 95% CI, 0.75–1.39) outcomes compared with those receiving immunochemotherapy with PF regimen (Fig. 5A-5C).

### Comparison of PFS and OS in patients with low PD-L1 expression levels by network meta-analysis

A network analysis focused on patients with low PD-L1 expression levels revealed that patients receiving immunochemotherapy with TP regimen demonstrated a statistically significant improvement in PFS (HR, 0.72; 95% CI, 0.56–0.93) compared with those receiving immunochemotherapy with PF regimen. Additionally, a trend toward improved OS associated with the TP chemotherapy regimen was observed (HR, 0.86; 95% CI, 0.63–1.16; Fig. 5D-5F).

## Discussion

To the best of our knowledge, the present study, which considered 8 phase III RCTs comparing immunochemotherapy versus chemotherapy alone as first-line systemic therapy for patients with ESCC represents the most comprehensive study of this type in this field. The results not only corroborated the findings of individual phase III RCTs regarding the survival benefits associated with the addition of anti-PD-1/PD-L1 therapy to chemotherapy but also revealed notable survival benefits across various patient subgroups, irrespective of PD-L1 expression levels (high or low) or chemotherapy regimens (PF or TP). The study findings are in line with a previous meta-analysis involving 5 phase III trials [30] and support the approval of anti-PD-1/PD-L1 therapy plus platinum-based chemotherapy as first-line systemic therapy for all patients with advanced ESCC by many health-related regulatory authorities worldwide.

Through a detailed comparison of the survival benefits across subgroups characterized by varying PD-L1 expression levels and different chemotherapy regimens, we demonstrated that the PFS and OS benefits derived from adding anti-PD-1/PD-L1 therapy to PF chemotherapy and to TP chemotherapy are generally comparable and that they are significant for patients with high PD-L1 expression levels. However, we observed that the PFS benefit is significantly more pronounced, and the OS benefit exhibits a trend toward improvement in patients with low PD-L1 expression levels receiving TP chemotherapy compared with those receiving PF chemotherapy. This heterogeneous impact of chemotherapy regimens on the survival benefits associated with the addition of anti-PD-1/PD-L1 therapy may help elucidate the discrepancies observed among various trials employing different chemotherapy regimens. For example, clinical trials adopting PF chemotherapy, such as CheckMate 648 [16–18] and KEYNOTE-590 [14, 15], have typically concluded that patients with low PD-L1 expression levels derive minimal benefit from the addition of PD-1 therapy to chemotherapy. Furthermore, clinical trials employing TP chemotherapy, such as JUPITER-06 and ORIENT-15 [22, 23], have revealed that the survival benefits are comparably significant between patients with low and high PD-L1 expression levels.

Two previous studies investigated the efficacy of different chemotherapy regimens in patients with advanced ESCC. These investigations indicated that TP chemotherapy was associated with improved PFS compared with PF chemotherapy; however, they did not demonstrate any significant differences in OS and ORR between the two chemotherapy regimens [32, 33]. Notably, these studies were retrospective analyses and meta-analyses, respectively, and both of which may be influenced by selection bias. In the RATIONALE-306 trial, a large-scale RCT that allowed investigators to select chemotherapy regimens, no significant difference in treatment efficacy was noted between the two chemotherapy subgroup control arms—TP alone (median PFS, 5.6 months; ORR, 42%) and PF/XP alone (median PFS, 5.5 months; ORR, 43%) [19]. Overall, these data do not indicate a significant difference in the treatment efficacies of TP chemotherapy and PF chemotherapy in patients with advanced ESCC.

In the present study, a statistically significant difference in PFS benefit was identified between patients treated with immunochemotherapy with TP regimen compared with those of patients who received immunochemotherapy with PF regimen. This finding is generally in line with those of previous reports. A retrospective study conducted in a single institution in China reported a significant difference in median PFS between these 2 regimens but no significant difference in OS [34]. Additionally, a previous meta-analysis that included 5 RCTs and 1 retrospective study demonstrated longer PFS and OS in patients receiving immunochemotherapy with TP regimen compared with those of patients receiving immunochemotherapy with PF regimen [32]. Studies have revealed that different chemotherapy agents might exert varying immunomodulatory effects [35, 36]. For example, one review highlighted that taxanes, compared with fluoropyrimidine, induced more immunogenic cell death, which may explain the improved therapeutic efficacy when combined with anti-PD-1/PD-L1 therapy [35].

In East Asia, the most common palliative chemotherapy regimen for advanced ESCC includes platinum—most commonly cisplatin—combined with a fluoropyrimidine or taxane [37]. In Netherlands, the most frequently used regimens are oxaliplatin plus fluoropyrimidine and carboplatin plus paclitaxel [38]. As the majority of patients enrolled in current study were from East Asia, particularly China, further research is needed to determine whether the findings can be generalized to Western populations.

Previous studies have demonstrated distinct adverse event (AE) profiles between TP and PF regimens in patients with EC undergoing neoadjuvant chemoradiotherapy (CRT) or definitive CRT [39]. These differences in AE patterns may influence treatment discontinuation rates and quality of life outcomes, which, however, cannot be assessed in the current study. In real-world clinical practice, the choice of chemotherapy regimen remains largely dependent on each patient’s comorbidities and overall clinical condition.

Our study has several limitations. First, PD-L1 expression was evaluated using various immunohistochemistry (IHC)-based assays in the included studies, with the assays involving different antibodies and scoring algorithms. However, a study reported that the concordance between different PD-L1 IHC monoclonal antibodies and scoring systems is acceptable when they are applied for the same ESCC tumor tissues [40]. Moreover, the definitions of high versus low PD-L1 expression adopted in the present study are consistent with those commonly adopted in previous meta-analyses of this nature [30, 31]. Second, in comparing the efficacy of different combination regimens, we generally assumed that all anti-PD-1/PD-L1 therapies were biologically equivalent and clinically similarly effective. Additionally, we assumed that patients treated with different chemotherapy regimens alone would have similar prognoses. Although the treatment efficacies of TP and XP/PF appeared comparable in the RATIONALE-306 study [19], no prospective RCT has yet to conduct a direct comparison of these chemotherapy regimens as first-line systemic therapy for ESCC. Therefore, differences may exist in the efficacy of PF versus TP chemotherapy regimens for patients with advanced ESCC, and this may have introduced bias into our analysis. Third, of the 4 included trials employing the TP chemotherapy regimen, only 2 (ESCORT-1st [20, 21] and JUPITER-06 [24]) reported survival analyses based on PD-L1 expression levels. The low number of patients for which such analyses were conducted may have reduced the robustness of our analysis and the strength of our conclusions. Forth, baseline characteristics with prognostic impact —such as performance status, age, comorbidities, and metastatic sites—might be imbalanced between different subgroups. OS may be influenced by imbalance baseline characteristics. Furthermore, only three of eight studies reported ORR data stratified by PD-L1 expression and chemotherapy regimen, limiting the depth of our current analysis. Further prospective studies with balanced prognostic factors are warranted to confirm the findings of current study.

## Conclusion

Our data suggest that the choice between TP and PF chemotherapy regimens may influence treatment outcomes, particularly in patients with low PD-L1 expression levels. Further RCTs are warranted to compare the efficacy, including the associated PFS and OS, of anti-PD-1/PD-L1 therapy combined with the TP regimen and anti-PD-1/PD-L1 therapy combined with the PF regimen in patients with advanced ESCC with low PD-L1 expression levels.

## Disclosures

Jhe-Cyuan Guo received travel expenses from Ipsen, Novartis, Ono Pharmaceutical, BeOne, and Merck Sharp & Dohme; honoraria from Ono Pharmaceutical, Merck Sharp & Dohme, Astellas Pharma, Roche/Genetech, Pfizer, Novartis, Ipsen, Bayer, and Merck Serono; had a consulting role for Ono Pharmaceutical, Merck Serono, and Astellas Pharma. Yu-Yun Shao received travel expenses from Roche and honoraria from Bayer, Bristol Myers Squibb, Eisai, Lilly, Ipsen, Merck, Ono Pharmaceutical, and Roche and research funding from Roche. Chih-Hung Hsu received travel expenses from Daiichi Sankyo and Roche; honoraria from Ono Pharmaceutical, Merck Sharp & Dohme, Bristol Myers Squibb, Roche, and Eisai; had a consulting role for Ono Pharmaceutical, Merck Serono, Bristol Myers Squibb, Roche/Genetech, and Daiichi Sankyo; research funding to institution from Ono Pharmaceutical, Merck Sharp & Dohme, Merck Serono, Taiho Pharmaceutical, Bristol Myers Squibb, BeiGene, Nucana, Johnson & Johnson, Roche/Genetech, NGM Pharmaceuticals, Eucure Biopharma, Surface Oncology, and Ipsen. Other authors had no conflict of interest.

## Supplementary Information

Below is the link to the electronic supplementary material.

Supplementary file1 (JPG 1218 KB)

Supplementary file2 (JPG 997 KB)

Supplementary file3 (DOCX 20 KB)

Supplementary file4 (DOCX 17 KB)

Supplementary file5 (DOCX 21 KB)

## Data Availability

The data is the property of the original trialists, with whom data sharing agreements would need to be reached.
