# Supplementary figures and images for: Does chemotherapy regimen matter for first-line immunochemotherapy in low PD-L1-expressing esophageal squamous cell carcinoma? A systemic review and meta-analysis
Source: Esophagus. 2025 Nov 10;23(1):25–36. doi: 10.1007/s10388-025-01167-y (PMC12832574; doi:10.1007/s10388-025-01167-y)

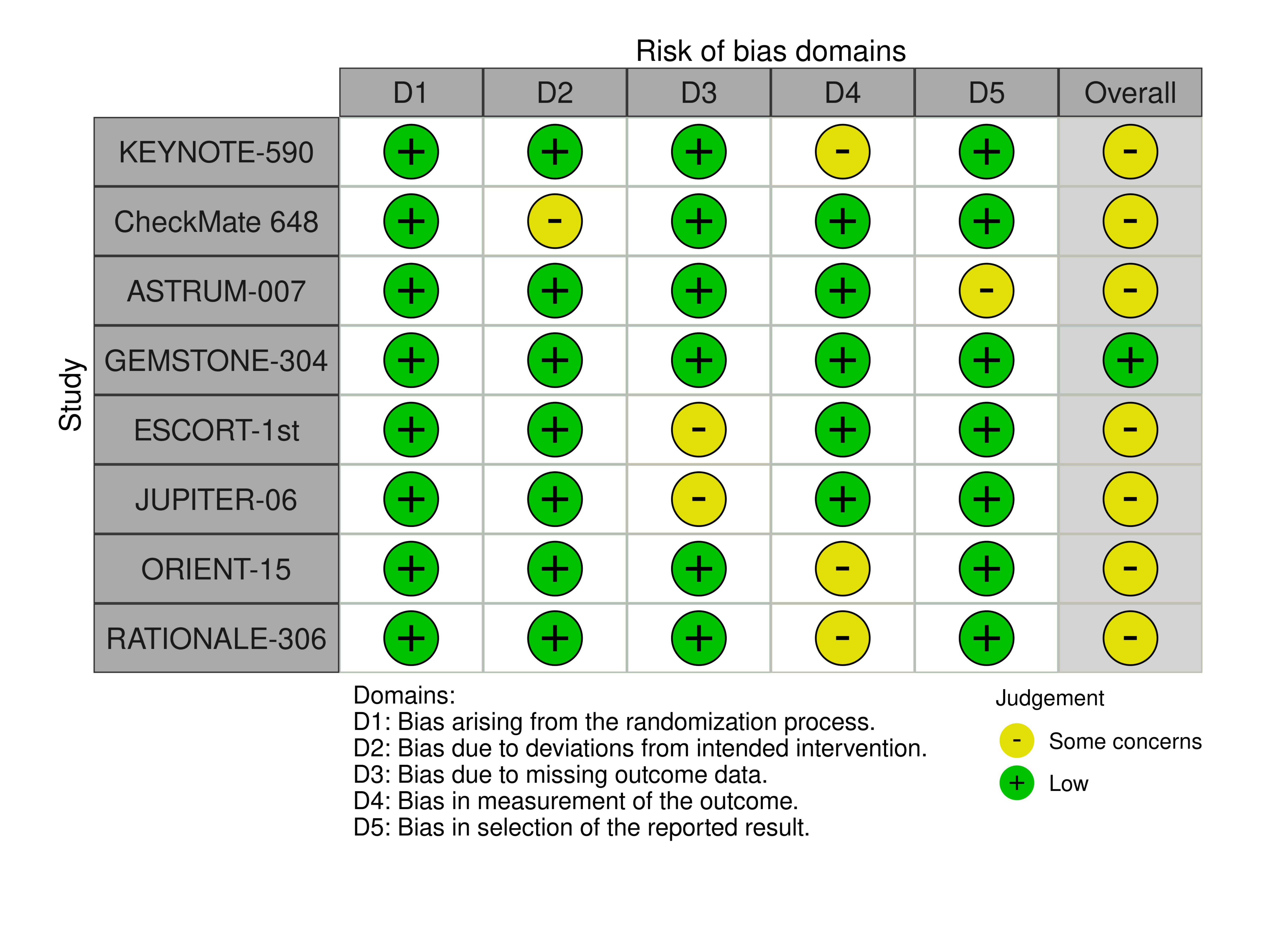

Supplement: Supplementary file 1 — Supplementary file1 (JPG 1218 KB) [file 10388_2025_1167_MOESM1_ESM.jpg]

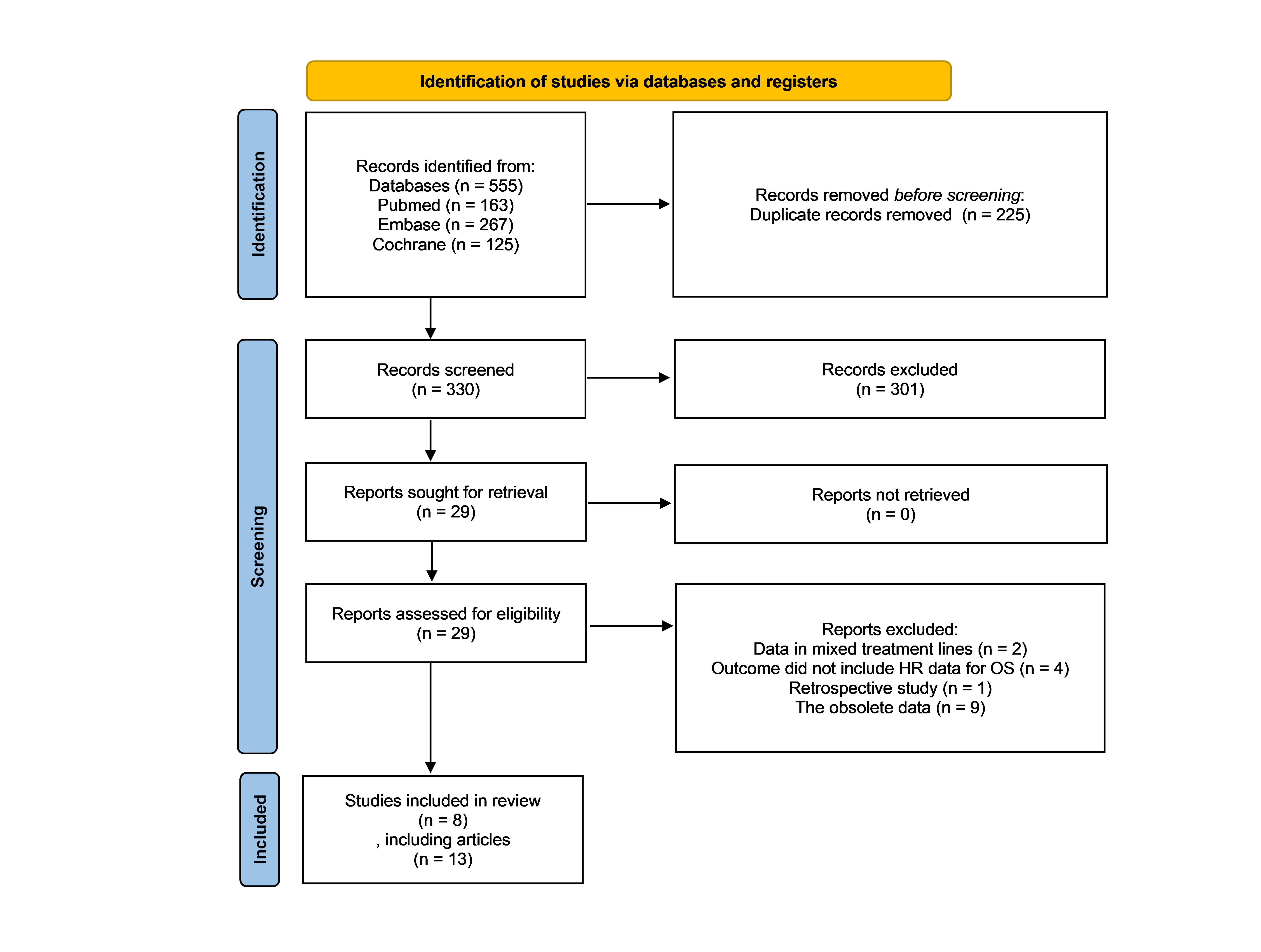

Supplement: Supplementary file 2 — Supplementary file2 (JPG 997 KB) [file 10388_2025_1167_MOESM2_ESM.jpg]
