# Supplementary material for: Does chemotherapy regimen matter for first-line immunochemotherapy in low PD-L1-expressing esophageal squamous cell carcinoma? A systemic review and meta-analysis
Source: Esophagus. 2025 Nov 10;23(1):25–36. doi: 10.1007/s10388-025-01167-y (PMC12832574; doi:10.1007/s10388-025-01167-y)
Supplement: Supplementary file 3 — Supplementary file3 (DOCX 20 KB) [file 10388_2025_1167_MOESM3_ESM.docx]

## Table S1. The number of subjects in the meta-analysis according to PD-L1 expression

| Figure 2A PFS | | | | Figure 2B OS | | | |
| --- | --- | --- | --- | --- | --- | --- | --- |
| Study | Exp.(N) | Control (N) | Total | Study | Exp.(N) | Control (N) | Total |
| Subgroup_High PD-L1 | | | | Subgroup_High PD-L1 | | | |
| ASTRUM-007 | 168 | 79 | 247 | ASTRUM-007 | 162 | 79 | 241 |
| ESCORT-1st | 166 | 163 | 329 | CheckMate 648 | NA | NA | 280 |
| GEMSTONE-304 | 154 | 78 | 232 | ESCORT-1st | 166 | 163 | 329 |
| JUPITER-06 | 115 | 97 | 212 | GEMSTONE-304 | 154 | 78 | 232 |
| KEYNOTE-590_SCC | 143 | 143 | 286 | JUPITER-06 | 115 | 97 | 212 |
| ORIENT-15 | 188 | 193 | 381 | KEYNOTE-590_SCC | 143 | 143 | 286 |
| CheckMate 648 | 158 | 157 | 315 | ORIENT-15 | 188 | 193 | 381 |
| RATIONALE-306 | 116 | 107 | 223 | RATIONALE-306 | 115 | 113 | 228 |
| Subtotal | 1208 | 1017 | 2225 | Subtotal | 1043 | 866 | 2189 |
|  |  |  |  |  |  |  |  |
| Subgroup_Low PD-L1 | | | | Subgroup_Low PD-L1 | | | |
| ASTRUM-007 | 206 | 104 | 310 | ASTRUM-007 | 206 | 104 | 310 |
| ESCORT-1st | 126 | 130 | 256 | CheckMate 648 | NA | NA | 329 |
| GEMSTONE-304 (CPS less than 1) | 41 | 21 | 62 | ESCORT-1st | 126 | 130 | 256 |
| GEMSTONE-304 (CPS between 1 to 10) | 163 | 83 | 246 | GEMSTONE-304 (CPS less than 1) | 41 | 21 | 62 |
| JUPITER-06 | 129 | 147 | 276 | GEMSTONE-304 (CPS between 1 to 10) | 163 | 83 | 246 |
| KEYNOTE-590_SCC | 121 | 126 | 247 | JUPITER-06 | 129 | 147 | 276 |
| ORIENT-15 | 139 | 139 | 278 | KEYNOTE-590_SCC | 121 | 126 | 247 |
| CheckMate 648 | 163 | 166 | 329 | ORIENT-15 | 139 | 139 | 278 |
| RATIONALE-306 | 151 | 168 | 319 | RATIONALE-306 | 149 | 160 | 309 |
| Subtotal | 1239 | 1084 | 2323 | Subtotal | 1074 | 910 | 2313 |
|  |  |  |  |  |  |  |  |
| Total | 2447 | 2101 | 4548 | Total | 2117 | 1776 | 4502 |

* NA: not available
