# Supplementary material for: Does chemotherapy regimen matter for first-line immunochemotherapy in low PD-L1-expressing esophageal squamous cell carcinoma? A systemic review and meta-analysis
Source: Esophagus. 2025 Nov 10;23(1):25–36. doi: 10.1007/s10388-025-01167-y (PMC12832574; doi:10.1007/s10388-025-01167-y)
Supplement: Supplementary file 4 — Supplementary file4 (DOCX 17 KB) [file 10388_2025_1167_MOESM4_ESM.docx]

## Table S2. The number of subjects in the meta-analysis according to chemotherapy regimen

| Figure 3A PFS and 3B OS | | | |
| --- | --- | --- | --- |
| Study | Exp.(N) | Control (N) | Total |
| Subgroup_PF | | | |
| ASTRUM-007 | 368 | 183 | 551 |
| CheckMate 648 | 321 | 324 | 645 |
| GEMSTONE-304 | 358 | 182 | 540 |
| KEYNOTE-590-SCC | 274 | 274 | 548 |
| ORIENT-15 (PF) | 20 | 23 | 43 |
| RATIONALE-306 (PF) | 147 | 146 | 293 |
| Subtotal | 1488 | 1132 | 2620 |
|  |  |  |  |
| Subgroup_TP | | | |
| ESCORT-1st | 298 | 298 | 596 |
| JUPITER-06 | 257 | 257 | 514 |
| ORIENT-15 (TP) | 307 | 309 | 616 |
| RATIONALE-306 (TP) | 179 | 177 | 356 |
| Subtotal | 1041 | 1041 | 2082 |
|  |  |  |  |
| Total | 2529 | 2173 | 4702 |

*PF: fluoropyrimidine (5-fluorouracil or capecitabine) + platinum (cisplatin or oxaliplatin); TP: paclitaxel + platinum (cisplatin or oxaliplatin)
