# Supplementary material for: Does chemotherapy regimen matter for first-line immunochemotherapy in low PD-L1-expressing esophageal squamous cell carcinoma? A systemic review and meta-analysis
Source: Esophagus. 2025 Nov 10;23(1):25–36. doi: 10.1007/s10388-025-01167-y (PMC12832574; doi:10.1007/s10388-025-01167-y)
Supplement: Supplementary file 5 — Supplementary file5 (DOCX 21 KB) [file 10388_2025_1167_MOESM5_ESM.docx]

## Table S3. The number of subjects in the meta-analysis according to PD-L1 expression and chemotherapy regimen

| Figure 4A PFS | | | | Figure 4B OS | | | |
| --- | --- | --- | --- | --- | --- | --- | --- |
| Study | Exp.(N) | Control (N) | Total | Study | Exp.(N) | Control (N) | Total |
| Subgroup_PF and high PD-L1 | | | | Subgroup_PF and high PD-L1 | | | |
| ASTRUM-007 | 168 | 79 | 247 | ASTRUM-007 | 162 | 79 | 241 |
| GEMSTONE-304 | 154 | 78 | 232 | GEMSTONE-304 | 154 | 78 | 232 |
| KEYNOTE-590_SCC | 143 | 143 | 286 | KEYNOTE-590_SCC | 143 | 143 | 286 |
| CheckMate 648 | 158 | 157 | 315 | CheckMate 648 | NA | NA | 280 |
| Subtotal | 623 | 457 | 1080 | Subtotal | 459 | 300 | 1039 |
|  |  |  |  |  |  |  |  |
| Subgroup_TP and high PD-L1 | | | | Subgroup_TP and high PD-L1 | | | |
| ESCORT-1st | 166 | 163 | 329 | ESCORT-1st | 166 | 163 | 329 |
| JUPITER-06 | 115 | 97 | 212 | JUPITER-06 | 115 | 97 | 212 |
| Subtotal | 281 | 260 | 541 | Subtotal | 281 | 260 | 541 |
|  |  |  |  |  | | | |
| Total | 904 | 717 | 1621 | Total | 740 | 560 | 1580 |
|  | | | | | | | |
| Figure 4C PFS | | | | Figure 4D OS | | | |
| Study | Exp.(N) | Control (N) | Total | Study | Exp.(N) | Control (N) | Total |
| Subgroup_PF and low PD-L1 | | | | Subgroup_PF and low PD-L1 | | | |
| ASTRUM-007 | 206 | 104 | 310 | ASTRUM-007 | 206 | 104 | 310 |
| GEMSTONE-304 (CPS less than 1) | 41 | 21 | 62 | GEMSTONE-304 (CPS less than 1) | 41 | 21 | 62 |
| GEMSTONE-304 (CPS between 1 to 10) | 163 | 83 | 246 | GEMSTONE-304 (CPS between 1 to 10) | 163 | 83 | 246 |
| KEYNOTE-590_SCC | 121 | 126 | 247 | KEYNOTE-590_SCC | 121 | 126 | 247 |
| CheckMate 648 | 163 | 166 | 329 | CheckMate 648 | NA | NA | 329 |
| Subtotal | 694 | 500 | 1194 | Subtotal | 531 | 334 | 1194 |
|  |  |  |  |  |  |  |  |
| Subgroup_TP and low PD-L1 | | | | Subgroup_TP and low PD-L1 | | | |
| ESCORT-1st | 126 | 130 | 256 | ESCORT-1st | 126 | 130 | 256 |
| JUPITER-06 | 129 | 147 | 276 | JUPITER-06 | 129 | 147 | 276 |
| Subtotal | 1643 | 1277 | 2920 | Subtotal | 255 | 277 | 532 |
|  |  |  |  |  | | | |
| Total | 2337 | 1777 | 4114 | Total | 786 | 611 | 1726 |

*PF: fluoropyrimidine (5-fluorouracil or capecitabine) + platinum (cisplatin or oxaliplatin); TP: paclitaxel + platinum (cisplatin or oxaliplatin)

**NA: not available
